# Developmental Profile of Psychiatric Risk Associated With Voltage-Gated Cation Channel Activity

**DOI:** 10.1016/j.biopsych.2021.03.009

**Published:** 2021-09-15

**Authors:** Nicholas E. Clifton, Leonardo Collado-Torres, Emily E. Burke, Antonio F. Pardiñas, Janet C. Harwood, Arianna Di Florio, James T.R. Walters, Michael J. Owen, Michael C. O’Donovan, Daniel R. Weinberger, Peter A. Holmans, Andrew E. Jaffe, Jeremy Hall

**Affiliations:** a Neuroscience and Mental Health Research Institute, Cardiff University, Cardiff, United Kingdom; b MRC Centre for Neuropsychiatric Genetics and Genomics, Division of Psychological Medicine and Clinical Neurosciences, Cardiff University, Cardiff, United Kingdom; c Lieber Institute for Brain Development, Johns Hopkins University Medical Campus, Baltimore, Maryland; d Centre for Computational Biology, Johns Hopkins University Medical Campus, Baltimore, Maryland; e Departments of Psychiatry, Neurology, Neuroscience and Genetic Medicine, Johns Hopkins School of Medicine, Baltimore, Maryland; f Department of Biostatistics, Johns Hopkins Bloomberg School of Public Health, Baltimore, Maryland

**Keywords:** Brain development, Dorsolateral prefrontal cortex, Genome-wide association studies, Psychiatric disorders, Transcriptome, Voltage-gated cation channel activity

## Abstract

**Background:**

Recent breakthroughs in psychiatric genetics have implicated biological pathways onto which genetic risk for psychiatric disorders converges. However, these studies do not reveal the developmental time point(s) at which these pathways are relevant.

**Methods:**

We aimed to determine the relationship between psychiatric risk and developmental gene expression relating to discrete biological pathways. We used postmortem RNA sequencing data (BrainSeq and BrainSpan) from brain tissue at multiple prenatal and postnatal time points, with summary statistics from recent genome-wide association studies of schizophrenia, bipolar disorder, and major depressive disorder. We prioritized gene sets for overall enrichment of association with each disorder and then tested the relationship between the association of their constituent genes with their relative expression at each developmental stage.

**Results:**

We observed relationships between the expression of genes involved in voltage-gated cation channel activity during early midfetal, adolescence, and early adulthood time points and association with schizophrenia and bipolar disorder, such that genes more strongly associated with these disorders had relatively low expression during early midfetal development and higher expression during adolescence and early adulthood. The relationship with schizophrenia was strongest for the subset of genes related to calcium channel activity, while for bipolar disorder, the relationship was distributed between calcium and potassium channel activity genes.

**Conclusions:**

Our results indicate periods during development when biological pathways related to the activity of calcium and potassium channels may be most vulnerable to the effects of genetic variants conferring risk for psychiatric disorders. Furthermore, they indicate key time points and potential targets for disorder-specific therapeutic interventions.

SEE COMMENTARY ON PAGE e31

Biological insight to psychiatric disorders has come from the identification of molecular pathways enriched for genetic association, determined by large cohort genome-wide studies. However, the expression of many genes varies as a function of age, and therefore, the relevance of genetically associated pathways likely varies across development.

Schizophrenia, bipolar disorder (BD), and major depressive disorder (MDD) show considerable heritability and share a substantial component of genetic risk (1, 2, 3, 4, 5, 6). Variance between the disorders may in part be attributed to differences in the degree of neurodevelopmental impairment (3,7,8). Many psychiatric symptoms first present during adolescence or in early adulthood (9), implying that pathophysiology emerges as the brain matures (10). However, there is substantial evidence that altered neurodevelopment during earlier prenatal or postnatal periods may contribute to some, if not all, psychiatric disorders (3,11, 12, 13, 14). Identifying the developmental stage at which particular biological pathways are most likely to contribute to risk for psychiatric disorders is therefore an important step toward understanding disease etiology and targeting new treatments.

Genetic association studies of schizophrenia have consistently demonstrated a convergence of genetic risk upon sets of genes with synaptic functions (15, 16, 17, 18, 19), including discrete signal transduction pathways such as voltage-gated calcium channel complexes and glutamate receptor complexes. The most recent genome-wide association studies (GWASs) of BD and MDD also highlight genes and pathways related to synaptic activity (20, 21, 22, 23). Hence, signaling complexes in the synaptic membrane represent strong candidates for psychiatric drug targeting.

Neurodevelopment is regulated by a program of tightly controlled gene expression (24,25). The majority of GWAS loci linked to psychiatric disorders are noncoding and most likely mediate risk by affecting gene expression (26, 27, 28, 29). Genes harboring risk for schizophrenia and BD are also among those most dynamically expressed across development, particularly in prefrontal cortical regions (30, 31, 32). However, the question of the developmental stage at which genes and gene pathways associated with major psychiatric diseases are primarily expressed remains largely unresolved, limiting our ability to reduce risk and target treatments.

By integrating developmental transcriptomic data of the human brain with GWAS data of genetic risk in schizophrenia, BD, and MDD, we aimed to identify time points at which gene sets implicated in risk for psychiatric disorders are most strongly expressed, in view of highlighting periods when key biological pathways may preferentially confer risk to these disorders.

## Methods and Materials

### Genotype Data

We obtained common variant summary statistics from published GWAS data. The schizophrenia sample (40,675 cases, 64,643 controls) (17) is a meta-analysis of a GWAS derived from UK cohorts of patients taking clozapine (11,260 cases, 24,542 controls) and an international Psychiatric Genomics Consortium (PGC) study (29,415 cases, 40,101 controls) (33). Case-control samples for BD were compiled by the PGC from 32 cohorts of European descent (20,352 cases, 31,358 controls) (21). Genotype data for MDD (135,458 cases, 344,901 controls) were derived from a PGC meta-analysis of 7 cohorts of European descent (22). For direct comparison of genetic factors contributing to schizophrenia and BD, we utilized additional summary statistics from a published GWAS of cases (23,585 schizophrenia, 15,270 BD) matched for ancestry and genotyping platform (34).

### Transcriptomic Data

Human brain transcriptomic data from across the life span were obtained from two sources. A primary dataset was derived from postmortem dorsolateral prefrontal cortex (DLPFC) of 336 individuals with no history of psychiatric condition, referred to as BrainSeq (31). Samples ranged in age from second trimester to 85 years (Table S1 in Supplement 2). Tissue acquisition, processing, and genotyping have been described previously (31,35). We obtained a second, smaller human developmental transcriptomic dataset from the Allen Institute BrainSpan Atlas (36,37), from which we retained data for DLPFC samples (*n* = 40) ranging from the first trimester to 40 years (Table S1 in Supplement 2). Sixteen samples from BrainSeq and BrainSpan originated from the same individuals; these were removed from BrainSeq to ensure independence. Raw sequencing reads from BrainSeq and BrainSpan were processed using the same software pipeline (38, 39, 40, 41). Gene counts were converted to reads per kilobase of transcript per million mapped reads (RPKM) values (calculated in relation to the number of gene-assigned reads).

### Gene Sets

Gene sets defined by biological pathways were curated from the Gene Ontology (GO) database (42) (downloaded January 13, 2020), excluding gene annotations with evidence codes NAS (non-traceable author statement), IEA (inferred from electronic annotation), or RCA (inferred from reviewed computational analysis). For primary analyses, gene sets containing fewer than 100 genes were excluded to minimize the effect of outliers. After filtering, 1766 gene sets remained (Table S2A in Supplement 2).

Further GO definitions were obtained from the SynGO database (43), consisting of manually curated gene annotations of synaptic function and location. After filtering for sets containing at least 100 genes, 19 sets remained (Table S4A in Supplement 2).

### Gene Set Association Analysis

Gene set association analysis was performed in MAGMA (version 1.08b) (44). Using GWAS summary statistics, single nucleotide polymorphisms with a minor allele frequency greater than 1% were annotated to genes, allowing for proximal regulatory regions (35 kb upstream, 10 kb downstream) (45). Gene-wide association statistics were calculated using the single nucleotide polymorphism–wise mean model with the 1000 Genomes European reference panel (46) to control for linkage disequilibrium. Gene set association was calculated using a background of all genes, conditional on the brain expression of each gene. Brain expression was defined as log_2_(RPKM + 0.5), where RPKM is the mean average across BrainSeq samples.

Primary analyses aimed to identify gene sets significantly enriched for GWAS association signal. Stepwise conditional analyses were applied to significantly (false discovery rate [FDR] < 0.05) (47) associated gene sets to identify a reduced number of independent sets that efficiently summarize the biological themes underlying the association. During this process, gene sets were repeatedly retested for genetic association, each time selecting the set with the highest effect size and adding that set to the conditional variables. On each iteration, gene sets that were no longer nominally significant (unadjusted *p* ≥ .05) were excluded. This procedure resulted in gene sets that may have small amounts of overlap but contribute substantially distinct genetic risk.

In secondary analyses, we tested the relative association of subsets compared with a larger set by adding the larger set to the model as a conditional variable. Comparisons of genetic association between nonoverlapping gene sets were performed using a *z* test of beta values.

### Developmental Gene Expression Scores

Transcriptomic data were divided into developmental stages (Table S1 in Supplement 2). Stages containing fewer than two samples in BrainSpan or BrainSeq were excluded for that dataset. Accordingly, two samples from BrainSeq were excluded (one early fetal, one late infancy). Late adulthood was not represented in BrainSpan. To permit comparison between genes and control for covariates, we constructed a score measuring the expression of each gene at each developmental stage relative to all other stages (30). Expression scores were calculated by fitting a linear regression model to each gene using limma (48) and extracting the *t* statistic for each developmental stage term:

$$Expression∼\overset{5}{\underset{i\;=\;1}{\sum }}snpPC_{i}\;+\;sex\;+\;developmental\;stage$$

Developmental stage is a binary variable indicating whether the sample is from that stage or not. We controlled for genetic ancestry by covarying for the first five principal components defining sample genotypes (snpPC).

### Expression-Association Relationships

For selected gene sets, we assessed the relationship between developmental stage gene expression and association with a disorder using MAGMA (version 1.08b) interaction analysis (49):

$$Z=\beta_{0}\;+\;B\beta_{B}\;+\;S_{1}\beta_{1}\;+\;S_{2}\beta_{2}\;+\;S_{12}\beta_{12}\;+\;\varepsilon$$

In this model, the interaction term $S_{12}$ is defined as the product of gene set membership $S_{1}$ and gene property $S_{2}$ terms. This method determined whether enrichment for genetic association in a gene set is stronger for genes with a higher expression score. Because of sensitivity to outlier effects in smaller sets, primary analyses were restricted to sets ≥ 100 genes (49). Interaction analyses were two-tailed, and statistical correction was performed for each gene set at each developmental stage using the Bonferroni method (*p*.adj). Analyses yielding significant interactions in one disorder were repeated as secondary analyses in the remaining disorders.

### Clustering of Developmental Expression Trajectories

To generalize expression analyses specific to each developmental stage to the complete expression trajectory across all stages, we partitioned gene sets of interest into subsets of genes with similar trajectories. Gene expression at each stage (mean RPKM) was scaled by subtracting the mean and dividing by the standard deviation. A suitable number of clusters for K-means clustering was identified using a scree plot. The procedure was run using 20 sets of random partitions, allowing for a maximum of 20 iterations. Gene set association analyses were used to test for enrichment of GWAS signal in gene clusters.

## Results

### Independently Associated Gene Ontologies

To select gene sets for downstream analyses, we performed gene set association analysis on a filtered list of 1766 GO-derived gene sets. After FDR correction, we observed 57 gene sets enriched for association with schizophrenia (Table S2A in Supplement 2) and 11 associated with BD (Table S2B in Supplement 2). No sets remained significantly associated with MDD (Table S2C in Supplement 2). Because of redundancy between GO terms, we used conditional analyses to select gene sets with independent associations. This procedure yielded six sets independently associated with schizophrenia (Table S2A in Supplement 2) and two sets independently associated with BD (Table S2B in Supplement 2). Genes related to voltage-gated cation channel activity (VG-cation) were enriched for association with schizophrenia (β = .43, FDR = 6.2 × 10^−4^) and BD (β = .32, FDR = 0.025), but not MDD (β = .14, FDR = 1.0). Of the 125 genes in the VG-cation set, only 1 gene, CACNA1C, surpassed gene-wide significance (*p* < 2.5 × 10^−6^) (50) for association with both schizophrenia and BD (Figure S1A in Supplement 1). Using a more exploratory threshold of gene-wide association (*p* < 1 × 10^−4^), we noted three further genes contributing risk to both disorders (Figure S1B–D in Supplement 1).

### Developmental Stage–Specific Relationships Between Gene Expression and Genetic Association Within Biological Pathways

**Figure 1:**
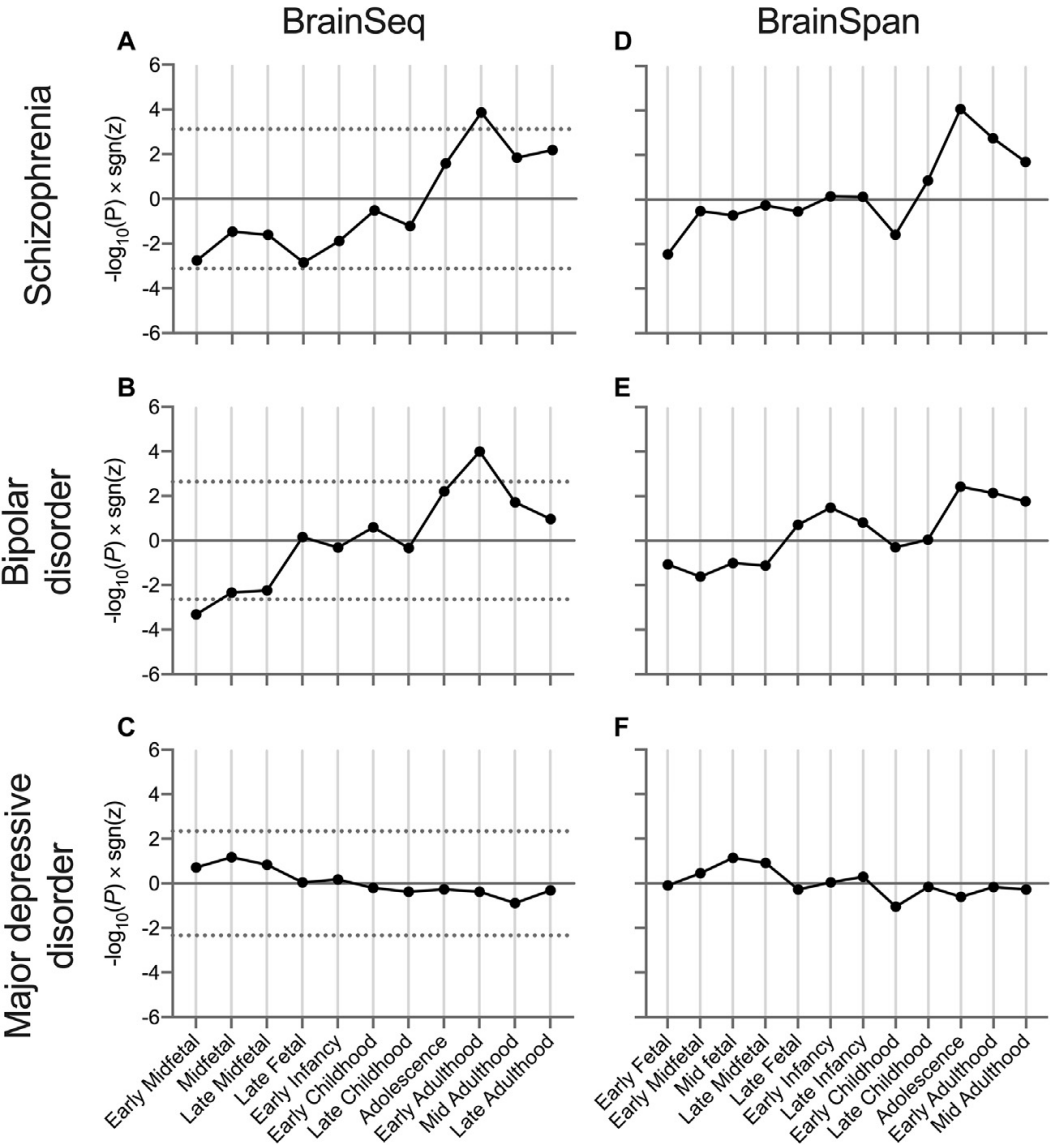
Developmental stage–specific relationships between the expression of voltage-gated cation channel activity genes and genetic association with psychiatric disorders. Shown is −log_10_(*P*)× sgn(*z*) from independent MAGMA interaction analyses of common variant association and gene expression scores derived from BrainSeq **(A–C)** or BrainSpan **(D–F)** at each developmental stage, compared with a background of all genes. Dotted lines indicate thresholds for statistical significance after correcting for analyses of all gene sets with independent association with the disorder and all stages (Table S3A, B in Supplement 2). Major depressive disorder analyses were corrected for the number of stages only. Developmental stages containing fewer than two samples in the dataset are not represented. early fetal, 8–9 pcw; early midfetal, 10–15 pcw; midfetal, 16 pcw; late midfetal, 17–23 pcw; late fetal, 24–37 pcw; early infancy, 0–5 months; late infancy, 6–11 months; early childhood, 1–5 years; late childhood, 6–12 years; adolescence, 13–19 years; early adulthood, 20–29 years; mid adulthood, 30–59 years; late adulthood, 60–100 years. pcw, postconceptual weeks.

We used BrainSeq transcriptomic data to determine whether enrichment for common variant association in gene sets with significant independent main effects is stronger for genes preferentially expressed during particular developmental stages. In analyses of genes annotated by VG-cation, we observed a significant positive interaction term for the relationship between expression during early adulthood and genetic association with both schizophrenia (β = .15, *p*.adj = .0090) (Table S3A in Supplement 2) and BD (β = .15, *p*.adj = .0022) (Table S3B in Supplement 2; Figure 1A, B). This indicates that during early adulthood, VG-cation genes more strongly associated with schizophrenia or BD have relatively high DLPFC expression than during other developmental stages, while those lacking association have relatively low expression (Figure S2 in Supplement 1). Conversely, during early midfetal development (10–15 postconceptual weeks), there was a negative relationship (indicating that genes more associated with the disorder have relatively low DLPFC expression and less associated genes have higher expression) between VG-cation expression and association with BD (β = −.047, *p*.adj = .011).

For comparison, we tested these temporal relationships with VG-cation expression in MDD genetic risk. VG-cation expression was not related to association with MDD at any developmental stage (Figure 1C).

We repeated analyses of developmental VG-cation expression using an independent transcriptomic dataset, BrainSpan. Overall, the pattern of preferential VG-cation expression across development was consistent between BrainSeq and BrainSpan (Figure 1D–F), such that early embryonic expression exhibited the strongest negative relationship with association with schizophrenia (early fetal: β = −.16, *p* = .0035; early midfetal: β = −.053, *p* = .30) and BD (early fetal: β = −.084, *p* = .086; early midfetal: β = −.10, *p* = .024), and positive relationships were observed during early adulthood (schizophrenia: β = .32, *p* = .0017; BD: β = .26, *p* = .0071). BrainSpan VG-cation gene expression during adolescence was also strongly positively related to association with schizophrenia (β = .32, *p* = 8.7 × 10^−5^) and BD (β = .21, *p* = .0038). Again, no relationships were observed between VG-cation temporal expression and MDD association.

Multiple synaptic pathways, including those related to voltage-gated ion channels, have been previously implicated in risk for psychiatric disorders (15). To assess the specificity of our findings, we studied an independent dataset of synaptic gene annotations for generalization. Using manually curated gene sets from the SynGO database (43), we repeated gene set association and developmental interaction analyses. From these sets, independent genetic associations were found for three gene sets with schizophrenia and three with BD (Table S4A, B in Supplement 2). None of these SynGO gene sets exhibited developmental patterns of expression that were linked to genetic association (Table S5A, B in Supplement 2).

### Partitioning Association Between Child Terms of Voltage-Gated Cation Channel Activity

The VG-cation term (125 genes) is composed of four nested “child” GO terms: voltage-gated potassium channel activity (VG-potassium, 80 genes), voltage-gated calcium channel activity (VG-calcium, 38 genes), NMDA glutamate receptor activity (7 genes), and voltage-gated proton channel activity (1 gene). To examine whether genetic association of VG-cation with schizophrenia and BD is further enriched in the smaller and more biologically specific “child” terms (excluding “voltage-gated proton channel activity” owing to size), we performed gene set analysis of these terms conditional on VG-cation. VG-calcium showed greater enrichment for association with schizophrenia than the full VG-cation set (Table 1). Conversely, association with BD was not further enriched within the “child” terms (Table 1), indicating even distribution across subtypes of VG-cation.

**Table 1:** The Relative Genetic Association of Voltage-Gated Cation Channel Activity “Child” Terms Compared With the Complete Gene Set in Two Psychiatric Disorders

		Schizophrenia			Bipolar Disorder			Schizophrenia vs. Bipolar Disorder	
Gene Set	*n*	β	*p* Value	*p.*conditional Value	β	*p* Value	*p.*conditional Value	β	*p* Value
Voltage-Gated Cation Channel Activity	125	.43	2.0 × 10^−6^	NA	.32	9.8 × 10^−5^	NA	.19	.0084
Voltage-Gated Calcium Channel Activity	38	.98	3.7 × 10^−9^	4.8 × 10^−5^	.41	.0064	.26	.15	.16
Voltage-Gated Potassium Channel Activity	80	.12	.15	1.0	.25	.0080	.87	.21	.015
NMDA Glutamate Receptor Activity	7	.90	.014	.12	.61	.046	.20	.12	.35

Shown is the output from gene set association analysis in MAGMA before (*p* value) and after (*p*.conditional value) conditioning on the full voltage-gated cation channel activity gene set. Also shown are results from contrasting the genetic contributions to the two disorders by gene set association analyses with summary statistics from schizophrenia vs. bipolar disorder genome-wide association study.

NA, not applicable.

Based on the differing contributions of VG-cation “child” terms to genetic association with schizophrenia and BD, we hypothesized that VG-calcium and VG-potassium are points of genetic divergence between the two disorders. We repeated the above gene set analyses using summary statistics from a schizophrenia versus BD GWAS (34). We found differential enrichment for genetic association with schizophrenia and BD in VG-cation genes (β = .19, *p* = .0084), which manifested significantly in VG-potassium (β = .21, *p* = .015) but not VG-calcium (β = .15, *p* = .16) (Table 1).

**Figure 2:**
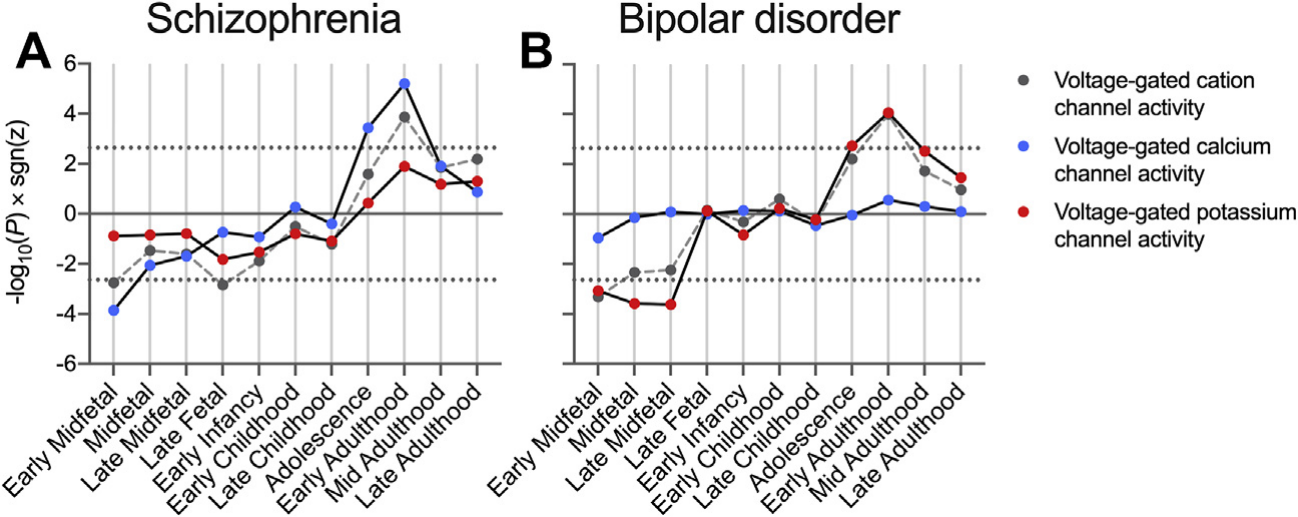
Comparison of expression-association relationships at 11 developmental stages within voltage-gated cation channel activity “child” terms. Shown is −log_10_(*P*) × sgn(*z*) from MAGMA interaction analyses of schizophrenia **(A)** or bipolar disorder **(B)** common variant association and BrainSeq-derived gene expression scores at each stage, compared with a background of all genes. Dotted lines indicate thresholds for statistical significance after correcting for analyses of two gene sets across 11 stages. GO:0022843 voltage-gated cation channel activity, *n* = 125; GO:0005245 voltage-gated calcium channel activity, *n* = 38; GO:0005249 voltage-gated potassium channel activity, *n* = 80.

We examined the contribution of VG-calcium and VG-potassium to the developmental patterns of preferential risk gene expression in VG-cation. Developmental relationships during early midfetal and early adulthood stages between gene expression and schizophrenia association were more prominent in VG-calcium than in VG-potassium genes (early midfetal: *z* test *p* = .039; early adulthood: *z* test *p* = .020) (Figure 2A). Conversely, these relationships in analyses of BD appeared more pronounced among VG-potassium genes (Figure 2B), although comparisons of effect sizes showed no significant differences at these stages (early midfetal: *z* test *p* = .49; early adulthood: *z* test *p* = .20), likely owing to the larger set size of VG-potassium than VG-calcium, giving greater significance for a given effect size. These analyses also strengthened the evidence that positive expression-association relationships begin in adolescence and continue into early adulthood.

Together, these results suggest that the VG-cation subset involved in calcium activity are further enriched for association with schizophrenia, with those more strongly associated being preferentially expressed during adolescence and early adulthood. In contrast, calcium and potassium subsets exhibit a more balanced association with BD and a greater dominance of preferential risk gene expression during early adulthood among potassium activity genes.

### Partitioning Association by Developmental Expression Trajectory

**Figure 3:**
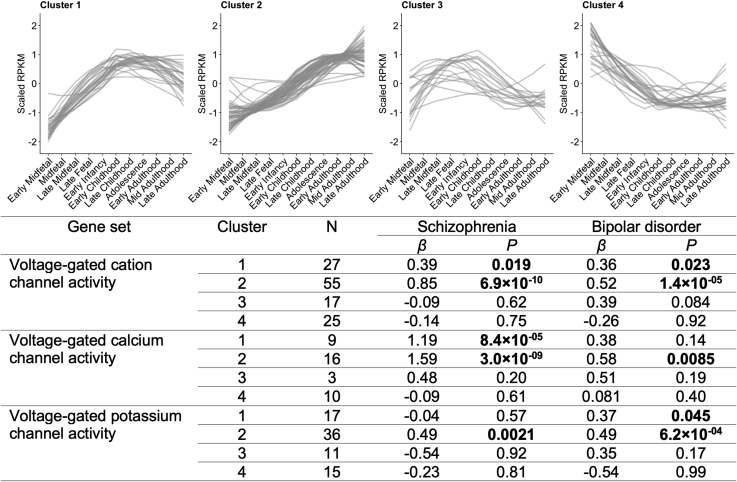
Clusters of developmental expression trajectories of voltage-gated cation channel activity genes and their enrichments for genome-wide association study association in schizophrenia and bipolar disorder. K-means clustering was used to divide genes based on their mean RPKM expression in BrainSeq across 11 developmental stages. Tabulated are results from schizophrenia and bipolar disorder gene set association analyses of each expression trajectory cluster, with separation into voltage-gated calcium channel activity and voltage-gated potassium channel activity “child” terms. RPKM, reads per kilobase of transcript per million mapped reads.

Our developmental stage–specific analyses suggest that VG-cation genes with low early midfetal and high adolescence/early adulthood expression are enriched for association with schizophrenia and BD. They also suggest that the schizophrenia association is stronger for the VG-calcium subset. To generalize these hypotheses across all developmental stages, we identified subsets of genes with similar expression trajectories and tested the distribution of genetic association. K-means clustering identified four broad expression trajectories in VG-cation genes (Figure 3; Figure S3 in Supplement 1; Table S6 in Supplement 2). Association with schizophrenia and BD was restricted to clusters 1 and 2, both of which contain genes with lower embryonic expression and higher expression in later life (Figure 3). Only cluster 2 (containing genes exhibiting later peak expression than cluster 1) harbored greater enrichment for association with schizophrenia and BD than VG-cation genes as a whole (schizophrenia: *p.*conditional = 3.6 × 10^−5^; BD: *p.*conditional = .010).

On splitting these clusters into VG-calcium and VG-potassium, we observed that the association of clusters 1 and 2 with schizophrenia was stronger among VG-calcium genes than among VG-potassium genes (cluster 1: *z* test *p* = .0019; cluster 2: *z* test *p* = 6.4 × 10^−4^), and only the VG-calcium subsets showed greater enrichment for association than VG-cation as a whole (VG-calcium cluster 1: *p.*conditional = .0062; VG-calcium cluster 2: *p.*conditional = 2.6 × 10^−6^; VG-potassium cluster 1: *p.*conditional = .99; VG-potassium cluster 2: *p.*conditional = .36). Association of the same clusters with BD was distributed between VG-calcium and VG-potassium subsets (cluster 1: *z* test *p* = .98; cluster 2: *z* test *p* = .75), which were not more enriched than the full VG-cation gene set (VG-calcium cluster 1: *p.*conditional = .42; VG-calcium cluster 2: *p.*conditional = .11; VG-potassium cluster 1: *p.*conditional = .39; VG-potassium cluster 2: *p.*conditional = .081).

## Discussion

Our study highlights biological convergence among common variants conferring risk of schizophrenia and BD in genes annotated by the ontological term “voltage-gated cation channel activity” (VG-cation) and identifies adolescence and early adulthood as periods of preferential expression for the most strongly associated members in both disorders. In schizophrenia, these temporal relationships derived predominantly from the calcium-related component of VG-cation, VG-calcium, whereas in BD, they were driven by both VG-calcium and VG-potassium components. Conversely, we observed no relationship between genetic association with MDD and preferential VG-cation gene expression at any developmental stage.

Enrichment for genetic association with schizophrenia, BD, and MDD in genes encoding voltage-gated calcium channel subunits has been consistently reported in primary GWAS (17,21,22), with additional genetic evidence supporting their involvement in schizophrenia from rare variants (19,51). While molecular abnormalities in calcium activity have historically been regarded as specific to BD (52, 53, 54), recent work suggests that alterations in cellular calcium may be comparable between schizophrenia and BD (55,56). The voltage-gated calcium channel family are signal transducers of electrical excitation in neurons. Through the induction of intracellular calcium transients, they couple membrane depolarization to signaling cascades including activity-dependent regulation of gene expression (57). In this way, voltage-gated calcium channels influence synaptic plasticity and are thought to be important drivers of learning and memory (58).

Unlike schizophrenia, genetic association with BD in VG-cation was distributed between calcium and potassium components. It is typically unfeasible to compare across disorders owing to differences in statistical power; however, we used summary statistics from a recent schizophrenia versus BD GWAS (34) to demonstrate that VG-potassium genes are differentially enriched for association with schizophrenia and BD. Voltage-gated potassium channels have been linked to BD through genetic studies previously (21,59, 60, 61, 62, 63), and this represents a divergence from schizophrenia and MDD, which lack evidence of conferring risk through potassium channels. Notably, the GO term “response to potassium ion” was the only pathway reported to be significantly differentially enriched for association in gene set analyses from the primary schizophrenia versus BD GWAS (34). The broad function of voltage-gated potassium channels is to control the threshold for action potentials and repolarize the membrane after firing (64). Similar to calcium channels, they have been shown to contribute to synaptic plasticity (65,66).

Our results suggest that adolescence and early adulthood are periods of peak preferential DLPFC expression of VG-cation genes associated with schizophrenia and BD. This lends to the idea that these developmental stages, which correspond to peak periods of symptom onset, may be more vulnerable to risk conferred through cation channels. Conversely, early midfetal development was characterized by comparably low expression of risk VG-cation genes, perhaps indicating a period of low vulnerability in this pathway. These conclusions are drawn from the assumption that peak expression relates to periods of high functional activity and the greatest impact of genetic variants. We cannot rule out, however, that change in the function of a gene expressed in relatively low abundance could mediate substantial risk.

More specifically, these periods of preferential gene expression in schizophrenia-associated VG-cation genes were characterized by more prominent relationships within the VG-calcium subset. Conversely, preferential expression of BD-associated VG-cation genes was agnostic to the cation subset. This discrepancy raises the possibility that VG-calcium genes, but not VG-potassium genes, contribute to an adolescence/early adulthood vulnerability to schizophrenia, while both subsets contribute to BD vulnerability during the same period.

Because of research bias and varying evidence quality, the database of GO annotations is undoubtedly incomplete. For example, the VG-cation GO term examined here does not include subsets related to sodium channel activity. Voltage-gated sodium channels have been linked to schizophrenia and autism in sequencing studies (51,67,68) and are involved in the pharmacodynamics of mood stabilizers used in the treatment of BD (69). Second, VG-calcium includes a small number of stargazin-like proteins, initially linked to voltage-gated calcium channels via homology, but with functions related to membrane expression of AMPA receptors, and are now collectively known as transmembrane AMPA receptor regulatory proteins (70).

Because of its manual curation, the SynGO database of synaptic annotations (43) is likely to have good functional accuracy. Although these data do not contain annotations of VG-cation genes, they include multiple gene sets annotated to synaptic pathways consistently implicated in psychiatric disorders (15, 16, 17, 18, 19). While some SynGO terms were enriched for association with schizophrenia or BD, the risk conferred was not related to periods of preferential gene expression. These results provide evidence that the developmental profile of schizophrenia and BD risk associated with GO VG-cation genes does not generalize to other synaptic pathways.

Our results reflect trends in gene sets, but expression patterns of individual genes or transcripts associated with psychiatric disorder may vary. For example, CACNA1C (encoding calcium channel Ca_V_1.2) contains a locus with genome-wide evidence for association with schizophrenia, BD, and MDD (17,21,71, 72, 73) and is the single-gene cause of Timothy syndrome, a disorder of multiorgan maldevelopment (74). CACNA1C is reported to reach peak brain expression in late fetal/early childhood development (75,76) and its forebrain deletion in embryonic, but not adult, mice models endophenotypes of psychiatric disorders (77). However, around 250 splice variants of CACNA1C have been reported (78), with varying expression profiles. Furthermore, it is still unclear which of the CACNA1C transcripts is associated with risk variants identified from GWAS. To better understand developmental vulnerability to genetic risk conferred through cation channels, the expression patterns of transcripts relevant to psychiatric disorders should be characterized independently of the gene-wide expression.

Our study employed transcriptomic data derived from the DLPFC, a region long thought to be affected in schizophrenia and mood disorders (79, 80, 81). Compared with other brain regions, the DLPFC is considered to be late maturing (82,83) and exhibits some distinct developmental gene expression trajectories (84,85). Hence, the relationships between genetic risk and developmental expression in other brain regions might be different or create age-specific vulnerabilities in other biological pathways that could account for the emergence of different psychiatric symptoms.

There is strong evidence that early developmental stages contribute to psychiatric pathophysiology, yet manifest as a disorder later in the life as the brain matures (11,80). Using similar methodology, our previous work (30) demonstrated that among all brain-expressed genes, there is a bias for those with stronger genetic association to be preferentially expressed in the prefrontal cortex during early midfetal development (schizophrenia) or early infancy (schizophrenia/BD). Other biological pathways associated with psychiatric disorders may be most vulnerable to genetic risk during prenatal or early postnatal life, but were not identified by this study.

Our findings have implications for the targeting of therapeutics. Psychiatric disorders are insufficiently treated, yet voltage-gated cation channels have been recognized as potential targets for new (or existing) compounds (66,86), and their druggability is an area of active research (87,88). Preferential expression of genetically associated genes during ages typical of diagnosis reinforces the suitability of voltage-gated cation channels as targets. Our results also indicate periods when therapeutic agents acting on such pathways may be most effective.

## Supplementary Material

Supplement 2

Key Resources Table
