# Supplementary material for: Developmental Profile of Psychiatric Risk Associated With Voltage-Gated Cation Channel Activity
Source: Biol Psychiatry. 2021 Sep 15;90(6):399–408. doi: 10.1016/j.biopsych.2021.03.009 (PMC8375582; doi:10.1016/j.biopsych.2021.03.009)
Supplement: Supplement 1 [file mmc1.pdf]

Developmental Profile of Psychiatric Risk Associated with Voltage-Gated Cation Channel Activity

Supplement 1

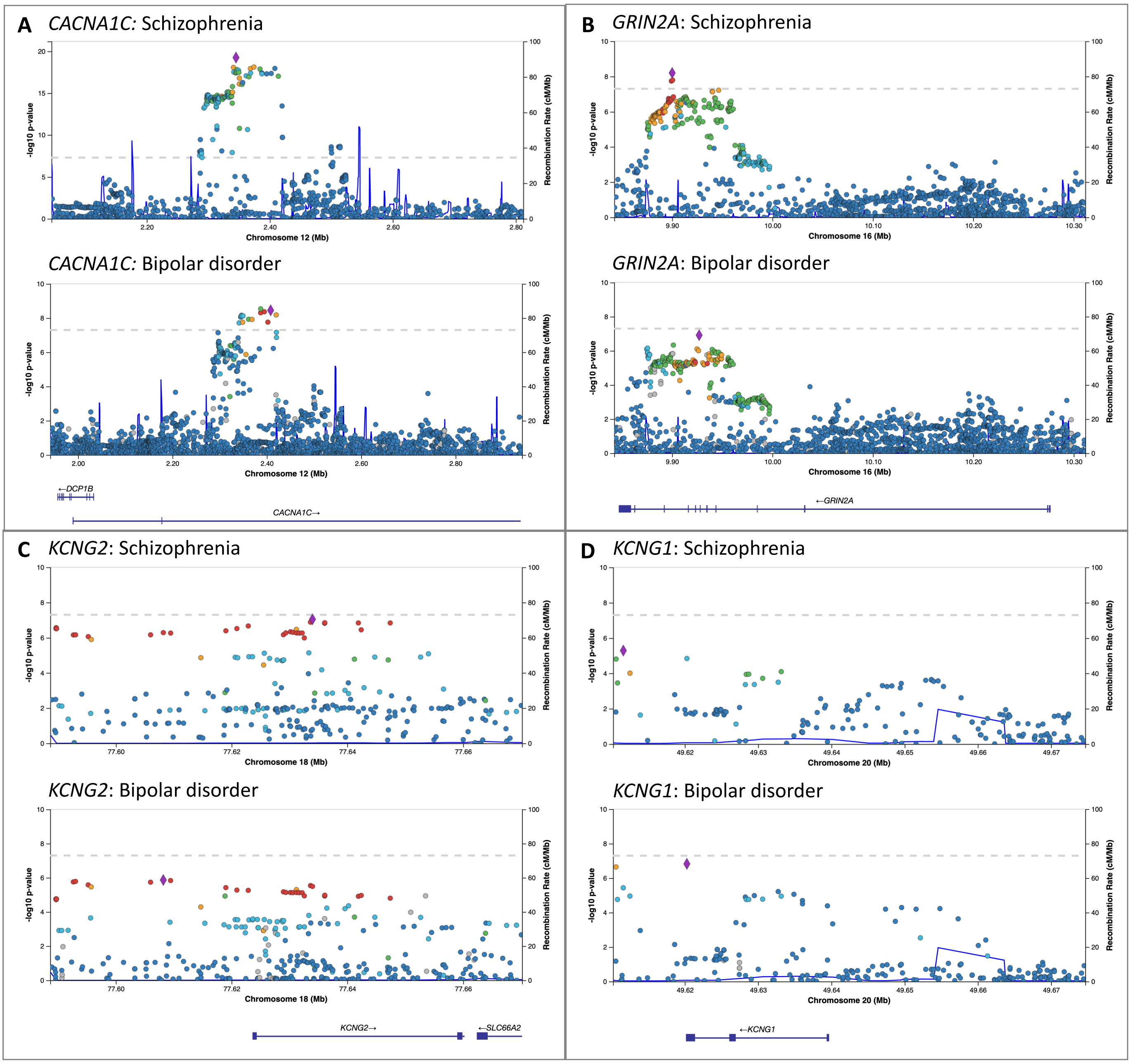

Figure S1 LocusZoom (1) plots of SNPs in four genes contributing risk to schizophrenia and bipolar disorder. Gene-wide *P*-values were calculated for *voltage-gated cation channel activity* (GO:0022843) genes using schizophrenia and bipolar disorder GWAS summary statistics. Shown are all genes from this set with gene-wide $P < 1\times10^{-4}$ in both disorders. Gene locations are indicated below each panel.

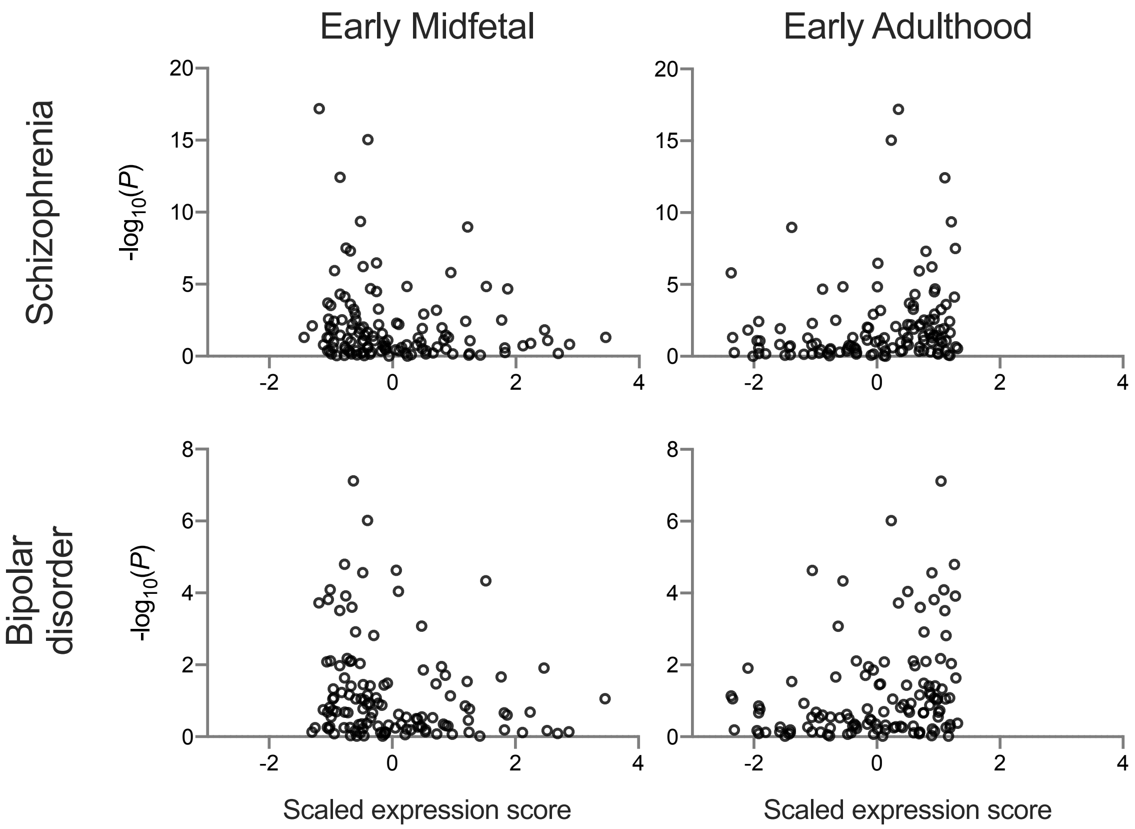

Figure S2 Comparisons of the relationship between GWAS association with schizophrenia or bipolar disorder and the relative expression of *voltage-gated cation channel activity* genes in BrainSeq during Early Midfetal and Early Adulthood developmental stages. Gene expression scores were scaled by subtracting the mean and dividing the standard deviation. Genetic association is represented by log converted gene-wide *P*-values from MAGMA analysis using the SNP-wise mean model, controlling for linkage disequilibrium.

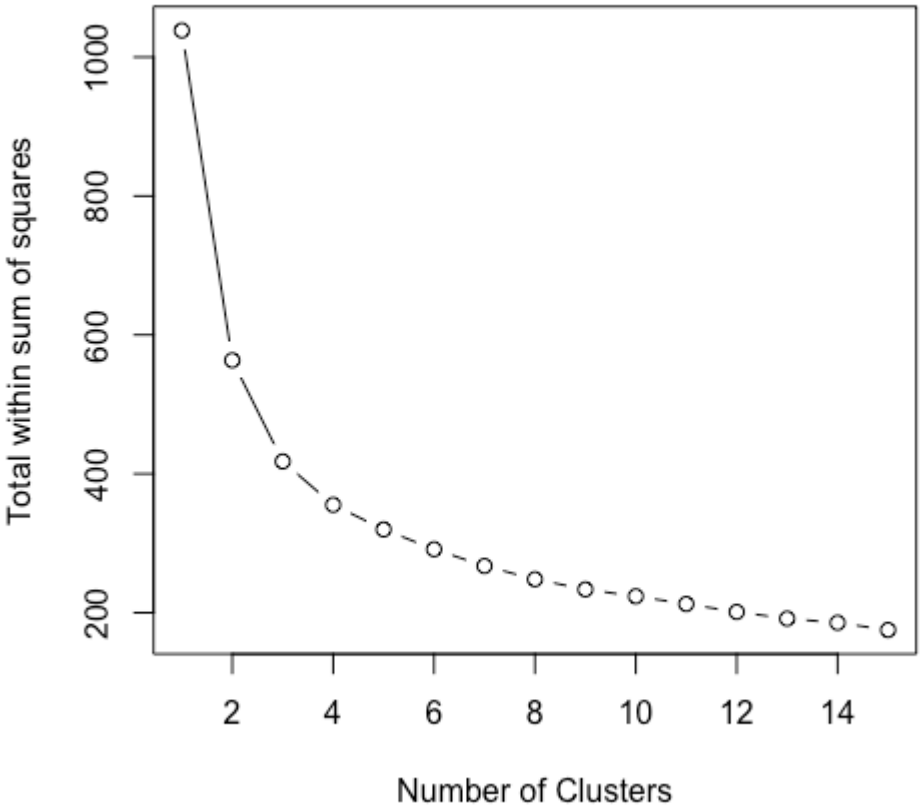

Figure S3 Scree plot from K-means clustering of *voltage-gated cation channel genes*. Clustering was performed for 1:15 centroids. Each run used 20 sets of random partitions, allowing for a maximum of 20 iterations per run.

Supplementary References

1. Pruim RJ, Welch RP, Sanna S, Teslovich TM, Chines PS, Gliedt TP, *et al.* (2010): LocusZoom: regional visualization of genome-wide association scan results. *Bioinformatics*. 26(18): 2336-7
